# Local delivery of FTY720 induces neutrophil activation through chemokine signaling in an oronasal fistula model

**DOI:** 10.1007/s40883-021-00208-z

**Published:** 2021-05-13

**Authors:** AM Amanso, TC Turner, A Kamalakar, SA Ballestas, LA Hymel, J Randall, R Johnston, RA Arthur, NJ Willett, EA Botchwey, SL Goudy

**Affiliations:** 1 Department of Otolaryngology, Emory University School of Medicine, 2015 Uppergate Drive, Atlanta, GA 30322 USA; 2 Coulter Department of Biomedical Engineering, Georgia Institute of Technology and Emory University, Atlanta, GA USA; 3 Petit Institute for Bioengineering and Biosciences, Georgia Institute of Technology, Atlanta, GA USA; 4 The Emory Integrated Computational Core, Emory University School of Medicine, Atlanta, GA USA; 5 Department of Orthopedics, Emory University School of Medicine, Atlanta, GA USA

**Keywords:** FTY720, Neutrophil, Oronasal fistula, Wound healing

## Abstract

**Purpose:**

Cleft palate repair surgeries lack a regenerative reconstructive option and, in many cases, develop complications including oronasal fistula (ONF). Our group has developed a novel murine phenocopy of ONF to study the oral cavity wound healing program. Using this model, our team previously identified that delivery of FTY720 on a nanofiber scaffold had a unique immunomodulatory effect directing macrophages and monocytes into a pro-regenerative state during ONF healing. Here, the objective of this study was to determine the effects of local biomaterial-based FTY720 delivery in the ONF model on the early bulk gene expression and neutrophil phenotypic response within the regenerating tissue.

**Methods:**

Using a mouse model of ONF formation, a palate defect was created and was treated with FTY720 nanofiber scaffolds or (blank) vehicle control nanofibers. At 1 and 3 days post-implantation, ONF oral mucosal tissue from the defect region was collected for RNA sequencing analysis or flow cytometry. For the RNA-seq expression profiling, intracellular pathways were assessed using the KEGG Pathway database and Gene Ontology (GO) Terms enrichment interactive graph. To assess the effects of FTY720 on different neutrophil subpopulations, flow cytometry data was analyzed using pseudotime analysis based on Spanning-tree Progression Analysis of Density-normalized Events (SPADE).

**Results:**

RNA sequencing analysis of palate mucosa injured tissue identified 669 genes that were differentially expressed (DE) during the first 3 days of ONF wound healing after local delivery of FTY720, including multiple genes in the sphingolipid signaling pathway. Evaluation of the DE genes at the KEGG Pathway database also identified the inflammatory immune response pathways (chemokine signaling, cytokine-cytokine receptor interaction, and leukocyte transendothelial migration), and the Gene Ontology enrichment analysis identified neutrophil chemotaxis and migration terms. SPADE dendrograms of CD11b^+^Ly6G^+^ neutrophils at both day 1 and day 3 post-injury showed significantly distinct subpopulations of neutrophils in oral mucosal defect tissue from the FTY720 scaffold treatment group compared to the vehicle control group (blank). Increased expression of CD88 and Vav1, among other genes, were found and staining of the ONF area demonstrated increased VAV1 staining in FTY720‐treated healing oral mucosa.

**Conclusion:**

Treatment of oral mucosal defects using FTY720 scaffolds is a promising new immunotherapy to improve healing outcomes and reducing ONF formation during cleft palate surgical repair. Local delivery of FTY720 nanofiber scaffolds during ONF healing significantly shifted early gene transcription associated with immune cell recruitment and modulation of the immune microenvironment results in distinct neutrophil subpopulations in the oral mucosal defect tissue that provides a critical shift toward pro-regenerative immune signaling.

**Supplementary Information:**

The online version contains supplementary material available at 10.1007/s40883-021-00208-z.

## Lay summary

Cleft palate patients (1:1000 live births) all undergo palate repair; 60% develop complications including oronasal fistula (ONF) formation which is associated with poor feeding and speech. Currently, cleft palate repair surgeries lack a regenerative reconstructive option, require multiple re-repairs due to ONF and increase cost and morbidity. FTY720, an active biolipid that activates sphingosine pathway, attracts pro-regenerative neutrophils, monocytes and macrophages to the oral wound site improving the ONF healing. The comprehensive understanding of the inflammatory signaling pathways controlled by FTY720 during ONF formation provide therapeutic targets for an effective immunomodulatory strategy to improve oral cavity wound healing, reducing ONF formation.

## Introduction

Abnormal craniofacial development can result in orofacial clefts, where there is continuity between the oral and nasal spaces. Cleft palate patients (1:1000 live births) all undergo surgical palate repair; however, 60% develop complications including oronasal fistula (ONF) formation. ONF formation leads to poor feeding, speech, and dental problems (1). Currently, cleft palate repair surgeries lack a regenerative reconstructive option, requiring multiple repairs (2). Persistence of the ONF despite re-repair occurs in 50% of patients, and the need for additional surgeries that require the use of local, regional, or free tissue transfer is higher in this patient population (3). To try to reduce the frequency of persistent ONF, some surgeons use a human dermal matrix; however, there is a risk for prion/HIV transmission, and the dermal matrix is only functioning as a barrier, not as a regenerative therapy (4). The high frequency and recurrence of ONF, along with the risks related to repeated anesthesia needed for re-repair, highlight the need for a regenerative approach to improve oral cavity during wound healing following cleft palate repair.

Our group has developed a novel murine phenocopy of ONF, and identified a unique immunomodulatory approach to direct oral mucosa repair to prevent ONF formation in a pro-regenerative program (5). FTY720 (Fingolimod), a sphingosine 1-phosphate (S1P) receptor modulator, is an FDA-approved drug to treat multiple sclerosis (6, 7). FTY720 acts as an agonist at four of the five S1P receptors, namely S1P1, S1P3, S1P4, and S1P5, and is involved in multiple signaling pathways such as Jnk (Jun N-terminal kinase), Akt (alpha serine/threonine-protein kinase), and Sphk (sphingosine kinase) (8). FTY720 is rapidly phosphorylated in vivo and the phosphorylated form is the biologically active molecule (9). Previous studies have shown that localized delivery of FTY720 from a nanofiber scaffold attracts pro-regenerative monocytes to cutaneous wound (10), specifically increasing the frequency of Ly6C^lo^ pro-regenerative monocytes associated with improved wound healing in soft tissues. FTY720 also increases the generation of pro-regenerative M2 macrophages in the site of the injury improving the healing outcomes and tissue regeneration (11). We recently published that the delivery of FTY720 nanofiber scaffolds also promotes rapid oral cavity wound healing and prevents ONF formation through similar mechanisms, while also inducing a more favorable interleukin expression (5). These results suggest that ONF wound healing following FTY720 nanofiber delivery induces a broad-based modulation of mucosal immunity that warrants investigation of additional immune subsets.

Although FTY720 regulates migration and signaling in multiple cell types, little is known about how FTY720 affects neutrophil function. Neutrophils play a critical role as the first responders of the immune system, and are the major innate immune cell that are recruited in the oral cavity (12, 13). There is also emerging evidence for the existence of heterogeneous neutrophil subsets within the larger neutrophil population that actively contribute to regenerative processes (14, 15). The objective of this study was to determine the effects of local biomaterial-based FTY720 delivery in the ONF model on the early bulk gene expression and neutrophil phenotypic response within the regenerating tissue. To study the effects of FTY720 on different neutrophil subpopulations, we performed RNA sequencing analysis of early gene expression changes (days 1 and 3) and pseudotime analysis based on Spanning-tree Progression Analysis of Density-normalized Events (SPADE) during ONF healing. These analyses allowed us to reconstruct complex cellular hierarchies of immune cell transitions in order to reveal rare cell states influenced by S1P receptor modulation that would have otherwise been overlooked (16).

## Material and methods

### Nanofiber scaffold production

Electrospun nanofiber scaffolds were fabricated using a 1:1 weight/weight ratio of Polycaprolactone (PCL, Sigma) and poly (lactic-co-glycolic-acid) (PLAGA, Lakeshore Biomaterials, Birmingham, AL). These polymers were then dissolved in a 1:3 volume ratio solution of methanol to chloroform to achieve an 18% polymer concentration for blank fibers, and 20% polymer concentration for FTY720-loaded fibers. For FTY720 loaded fibers, drug was added into the solution at a 1:200 drug:polymer weight ratio. The solution was then agitated for 2 + hours until the polymer was fully dissolved and then 2 mL of polymer solution was loaded into a 3 mL syringe with a 10 mm diameter. Electrospinning was performed at a flow rate of 1 mL/hr, at applied voltage of 19 kV for both blank and FTY720 fibers (17, 18). The working distance was 12 cm for FTY720 fibers and 10 cm for blank fibers. After 2 mL of polymer was spun, fibers were wrapped in low-binding plastic folders and stored at -20 °C. Our group had published a release study of FTY720 kinetics for the nanofiber scaffolds, where the majority of the drug is released during the first 24 hours post-injury (5). These polymer fiber sheets were then made into 1.5 mm diameter scaffolds using a biopsy punch.

### Oronasal fistula murine model

All *in vivo* experiments were performed using procedural guidelines with appropriate approvals from the Institutional Animal Care and Use Committee of Emory University. C57BL/6 mice (The Jackson laboratory, 000664) were used. All mice were 8–12-week females and had a weight of 20–25 g. To create an ONF, the mice were anesthetized with Isoflurane 3% and Ketamine/Xylazine 100 mg/kg/10 mg/kg, and a retractor was used to open their mouth (5). Following the anesthesia, a 1.5-mm full-thickness hard palate mucosal injury was created in the midline using an ophthalmologic cautery. Nanofiber scaffold implantation following the injury was secured using TA-5 veterinary tissue glue. All mice had 1.5-mm oronasal fistulas verified using a 1.5-mm biopsy punch as a measuring tool. In the event of creation of an oronasal fistula bigger than 1.5 mm, animals were eliminated from the study.

### RNA sequencing

Oral mucosal tissues were harvested and whole-genome RNA was isolated using the Qiagen RNeasy kit (Qiagen, 74106) according to the manufacturer’s protocols. Quality Control (QC) was done using a bioanalyzer to determine the RNA Integrity Number (RIN) of the samples. Thereafter, a New England Biolab’s (NEB) mRNA isolation module in conjunction with their Ultra II RNA directional kit (NEB, E7760) was used to generate sequencing libraries. QC was then performed on these libraries using the bioanalyzer, and the library was quantified using fluorometric methods. Paired-end 75 base pairs (PE75) sequencing was performed on the Illumina NEXTSeq instrument to obtain a sequencing depth of 30000 reads per sample. The transcripts obtained were aligned and annotated by comparing them to the genome-wide mouse database (mm9) along with elimination of duplicate reads, using the strand next-generation sequencing (NGS) application. The RNA levels were calculated in reads per kilobase per million mapped reads (RPKM). Genes expressed at >1.5 RPKM were included in further analyses (19). Gene quantification was done using HTSeq-count (20). Differential expression analysis was conducted using DESeq2 (21). Genes with mean-normalized counts of less than 4 were removed from analysis. Raw *p*-values were transformed using the Benjamini-Hochberg correction to account for multiple hypothesis testing. Genes considered significantly differentially expressed are those with adjusted *p*-values, also called False Discovery Rates (or FDR) controlled at < 0.05. Volcano plots were generated using EnhancedVolcano, and heat maps were generated using GraphPad Prizm.

### Gene Ontology and KEGG pathway analysis

Gene Ontology (GO) analysis was performed using https://tools.dice-database.org/GOnet/ (22). The application produced interactive visualizations of the GO analysis results, showing the hierarchy of the terms and retains relationships between terms and genes/proteins. The cell signaling pathway analysis was performed using DAVID Bioinformatics Resources 6.8 (https://david.ncifcrf.gov/) (23, 24). The pathways were extracted from the KEGG (Kyoto Encyclopedia of Genes and Genomes) database (25). GO annotations and KEGG pathways reported were obtained from a list of genes from the RNA sequencing analysis of FTY720 versus (blank) vehicle control nanofibers with *q*≤0.05.

### Confocal microscopy analysis of VAV1 and CD88

Mice were euthanized at postoperative day 3, and palate tissue was harvested and fixed with 10% buffered formalin. Paraffin-embedded tissues were cut in 10 μm sections and stained with anti-VAV1/Alexa568 (Invitrogen, cat# MA531488) or anti-CD88/Alexa488 (Biorad, cat# MCA2456GA). Images were acquired using the ×10 objective on an Olympus F1000 microscope.

### Flow cytometry

For analysis of immune infiltrate, oral mucosal tissues were harvested and digested in 1 mg/ml collagenase I (Sigma) for 45 min at 37 °C. The digested palate mucosa was filtered through a cell strainer to obtain a single-cell suspension. Single-cell suspensions from palatal samples were stained for live cells using Zombie NIR (Biolegend) dyes in cell-culture grade PBS per manufacturer instructions. Cells were then stained with cell phenotyping antibodies in a 1:1 vol ratio of 3% FBS and Brilliant Stain Buffer (BD Biosciences) according to standard procedures and analyzed on a FACS AriaIIIu flow cytometer (BD Biosciences). The following antibodies were used for cell phenotyping: Zombie NIR Fixable Viability Kit (BioLegend), BV606-conjugated CD45 (BioLegend), PE-conjugated MERTK (BioLegend), PerCP-Cy5.5-conjugated CD64 (BioLegend), APC-conjugated Ly6C (BioLegend), PE-Cy7-conjugated Ly6G (BioLegend), FITC-conjugated CD11b (BioLegend), and APC-Cy7-conjugated CD206 (BioLegend).

### Spade analysis

Spanning-tree progression analysis of density-normalized events (SPADE) is a nonlinear dimensionality reduction method that orders the progress of immune cell subsets through biological processes based on molecular marker similarities (26). SPADE creates a tree dendrogram where nodes represent clusters of cells with similar marker expression. The size and color intensity of each node are determined relative to the number of cells present and the median marker expression. The frequency of defined cells approximates the cell’s position as discrete nodes in an ordered process. The inferred order is referred to as “pseudotimes” and is used here to understand regulatory trajectories of immune cell infiltration. SPADE was performed through MATLAB and using source code made available from GT/Emory. The MATLAB-based SPADE software automatically generates the tree from flow cytometry raw data files by performing density-dependent down-sampling agglomerative clustering, linking clusters with a minimum spanning-tree algorithm, and up-sampling based on user input.

## Results

### FTY720-induced differential gene expression following murine ONF creation

**Fig. 1:**
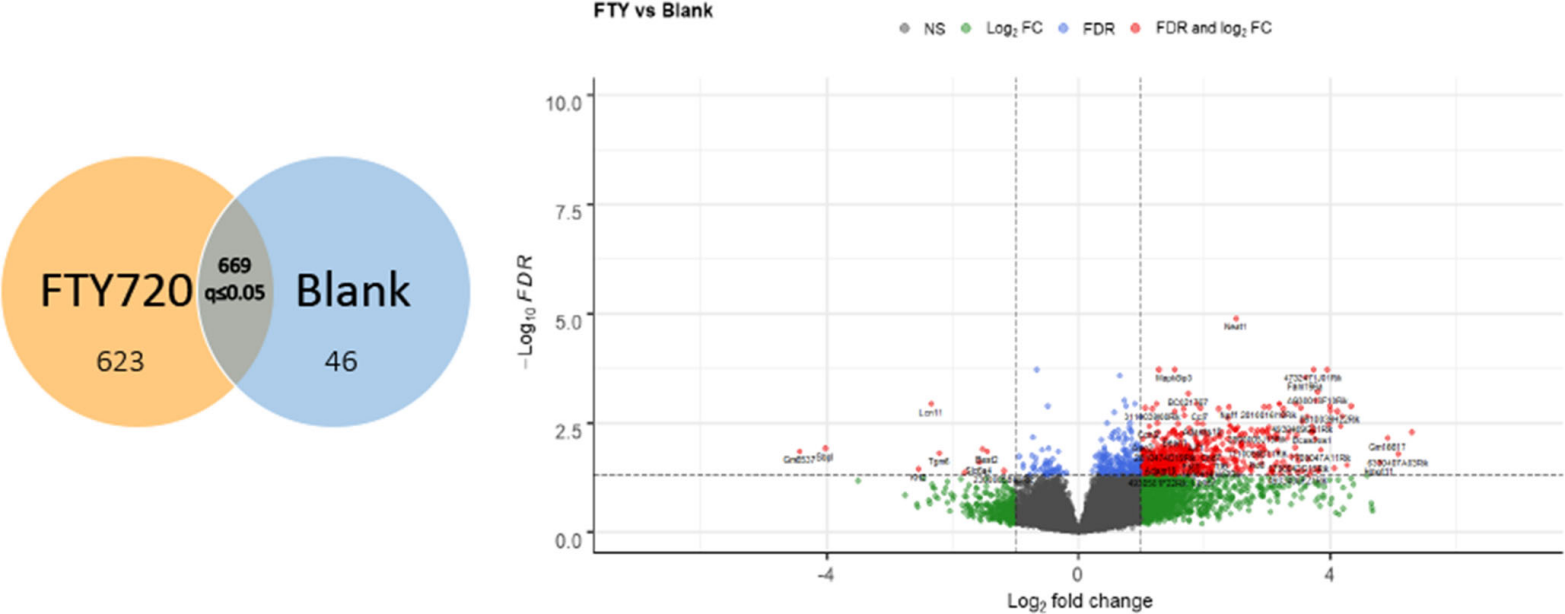
RNA sequencing analysis in the mucosa palate after local delivery of FTY720 in oral wound injury. ONF was created in 11 mice and scaffolds with or without FTY720 were added at the site of the injury for a local drug delivery. From a total of 11 animals, 6 animals were treated with blank scaffold (with no drug added) and 5 were treated with scaffolds loaded with FTY720. RNA sequencing analysis were performed in the mucosa of the hard palate. The sequencing revealed 669 differential expressed genes (DE). The yellow circle indicated that 623 genes were upregulated by FTY720, and the blue circle is the 46 genes downregulated by FTY720. The volcano plot revealed many more genes are significantly upregulated than downregulated by FTY720 considering *p* adjusted *q*≤0.05

Oral cavity wound healing following oral cavity mucosal injury, from surgery or trauma, occurs in a bacteria-rich environment that sustains continual trauma due to mastication. Despite these challenges, oral cavity wound healing typically occurs quickly and in a scarless fashion (27). However, failure of oral cavity wound healing following cleft palate repair in children leads to oronasal fistula (ONF) formation in up to 60% of patients. Using a mouse model of ONF formation (5), we implanted FTY720 nanofiber scaffolds into the oral defect and performed RNA sequencing analysis on healing oral mucosal tissues at day 1 and day 3 following injury. Six animals were treated with (blank) vehicle control nanofibers (with no drug added) and 6 were treated with scaffolds loaded with FTY720 (one of the FTY720 mice died and was excluded from analysis). After 1 and 3 days post-surgery, the mucosa from the defect region of the hard palate was harvested, and the RNA was extracted and sequenced. Sequencing was performed on the Illumina NEXTSeq instrument to obtain a sequencing depth of 30000 reads per sample. To perform the analysis of differentially expressed genes from FTY720 versus blank treated palate, we pooled all of the FTY720 scaffold mucosal samples (day 1 and 3) and compared them to the blank scaffolds (day 1 and 3); the False Discovery Rate or adjust *p*-value (*q*) was calculated. From a total of 24421 variables, 3513 of genes were filtered for mean normalized counts <2. The volcano plot showed that more genes were significantly upregulated than downregulated (Fig. 1). Fold change cut-off was set at 1.0; FDR was 0.05. 669 genes had an adjusted *p* or *q*≤0.05, 623 were upregulated, and 46 genes were downregulated. Thirty-three genes were removed as outliers by Cook’s distance criteria. To determine the effect of FTY720 scaffold delivery, the 669 differentially expressed genes were analyzed using the KEGG database and we identified that 53 pathways were impacted (Supplemental Table 1).

### Activation of Sphingolipid Signaling by FTY720 scaffolds during ONF wound healing

**Fig. 2:**
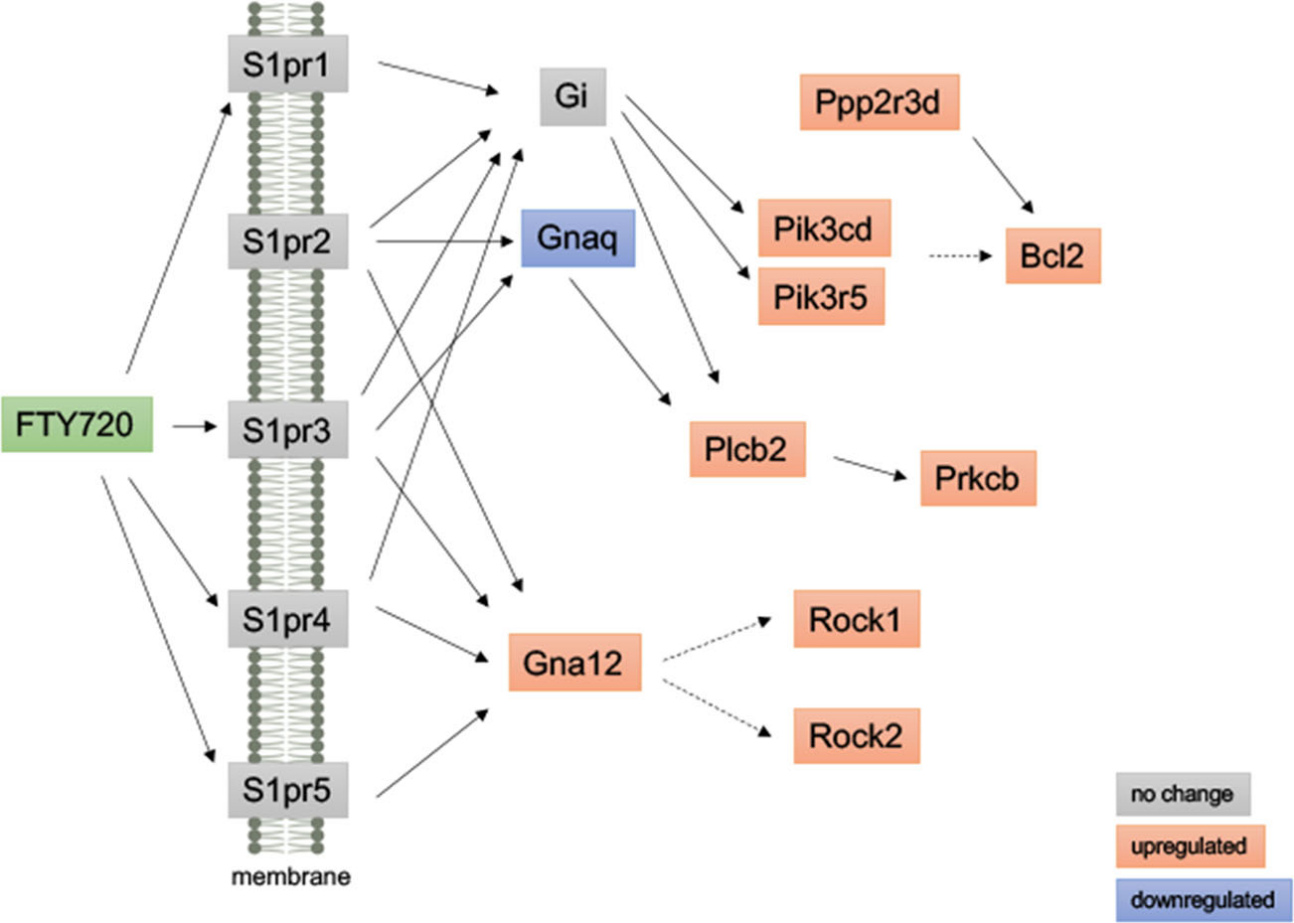
Activation of sphingolipid signaling by FTY720 during wound healing after palatal injury. Representative graph of the sphingolipid signaling pathway. Pathway analysis of RNA sequencing from palate mucosa treated immediately after injury with or without FTY720 scaffolds revealed that the sphingolipid signaling pathway (KEGG Database-map04071) was activated by the FTY720 local delivery. In the graph, the genes annotated in the orange box indicate that they were upregulated and the genes in the blue box were downregulated by FTY720

FTY720 mediates its effects by modulating shingosine-1-phosphate (S1P) receptors and altering intracellular sphingolipid (8). In this study, the sphingolipid pathway was found to be one of the 53 pathways affected by FTY720 scaffold delivery on KEGG analysis: signaling transduction map04071 - sphingolipid signaling pathway (Fig. 2). Although S1P receptor gene expression was not changed following the delivery of FTY720 scaffolds, evaluation of the downstream signaling pathway identified 10 genes downstream of the S1PR were differentially regulated, with 9 genes upregulated (Bcl2, Gna12, Pik3cd, Pik3r5, Plcb2, Prkcb, Ppp2r3d, Rock1, and Rock2) and 1 gene downregulated (Gnaq). The heatmap and log2foldchange of differentially expressed genes are shown in Figs. 9 and 10.

### FTY720 scaffolds induces the expression of chemokine signaling and leukocyte migration genes during ONF healing

**Fig. 3:**
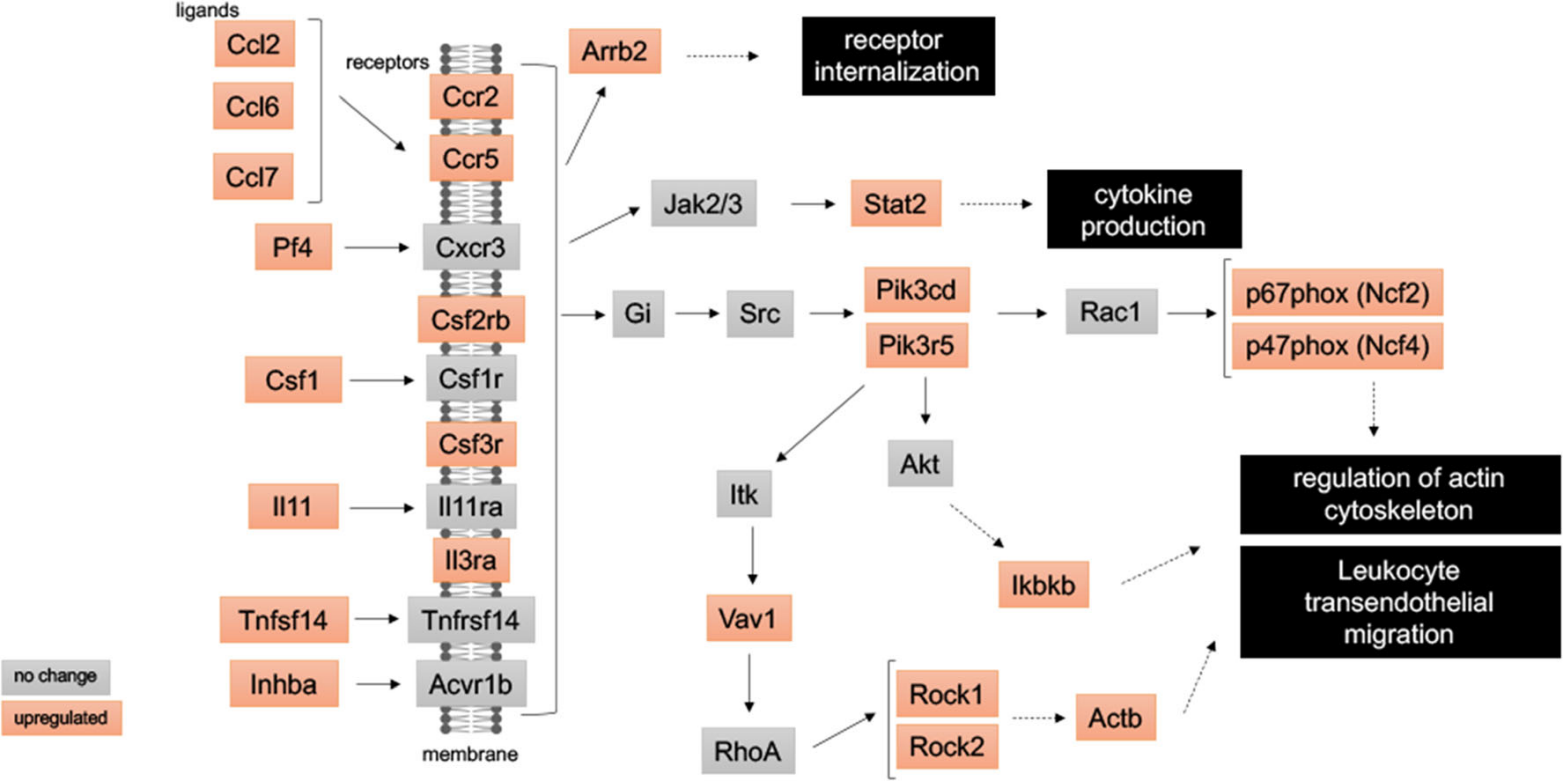
FTY720 induces chemokine signaling and leukocyte migration during wound healing after palatal injury. Representative graph of the immune system pathways induced by FTY720 delivery at wounded palate. Pathway analysis RNA sequencing from palate mucosa treated immediately after injury with or without FTY720 scaffolds revealed that FTY720 delivery activated the following pathways: map04062-Chemokine signaling pathway, map04060-Cytokine-cytokine receptor interaction, and map04670-Leukocyte transendothelial migration. In the graph, the genes annotated in the orange box indicate that they were upregulated by FTY720 treatment

KEGG analysis of differentially expressed genes from the mucosal defect tissue treated with FTY720 scaffolds identified significant changes in chemokine signaling and leukocyte migration in the first 3 days after injury (Supplemental Table 1). Evaluation of the KEGG analysis after FTY720 scaffold delivery identified increased expression of the inflammatory immune response pathways: 1-map04062 Chemokine signaling pathway, 2-map04060 Cytokine-cytokine receptor interaction and 3-map04670 leukocyte transendothelial migration (Fig. 3). In total, 27 genes from these pathways were upregulated by FTY720 scaffold treatment: Actb, Arrb2, Ccl2, Ccl7, Ccl9, Ccr2, Ccr5, Csf1, Csf2rb, Csf3r, Ikbkb, Il3ra, Il11, Inhba, Itgam, Ncf2, Ncf4, Pf4, Pik3cd, Pik3r5, Plcb2, Prkcb, Rock1, Rock2, Stat2, Tnfsf14, and Vav1. The heatmap and log2foldchange are represented at the Figs. 9 and 10. The cellular responses induced by these pathways are diverse, including (1) innate as well as adaptive inflammatory host defenses; (2) recruitment of leukocytes to the site of inflammation; (3) cell differentiation, cell death, and angiogenesis; and 4) repair processes aimed at the restoration of homeostasis (28–31).

### FTY720 scaffold delivery induces expression of neutrophil chemotaxis and migration genes in ONF model

**Fig. 4:**
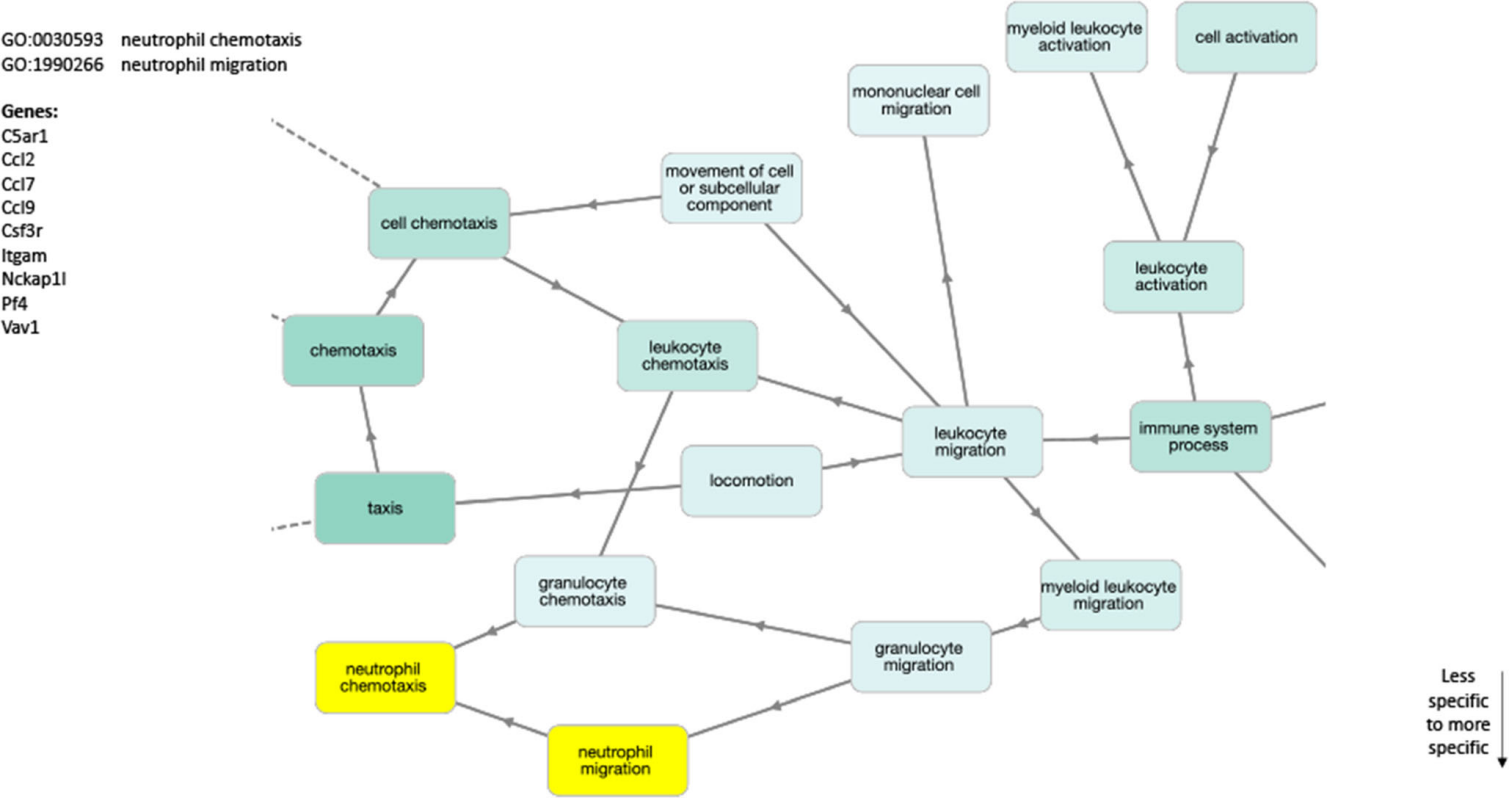
FTY720 induces neutrophil chemotaxis and migration after palatal injury. Gene Ontology analysis graph. GOterm analysis of RNA sequencing from palate mucosa treated immediately after injury with or without FTY720 scaffolds showing terms related with Leukocytes. Highlighted in yellow are the correspondent nodes from Neutrophils GOterms, GO:0030593-Neutrophil Chemotaxis and GO:1990266-Neutrophil Migration. In the left on the graph is the list of genes present in these nodes

Neutrophils are the leukocyte population most commonly found in the initial stage of wound healing (32). To determine the impact of FTY720 scaffold delivery on neutrophils, the 669 DE genes were analyzed for Gene Onthology (GO) using GOnet, open-source web-application approach for reviewing GO annotations and searching for enrichment patterns (22). GOnet was used to construct an interactive graph containing GO terms and genes. These graphs convey the hierarchical structure of the GO terms and connect GO terms to the genes used in the entry. The hierarchy between terms implies the possibility to propagate annotation, so the same gene present in one node may be also annotated by all of its ancestors. The Gene Ontology analysis, using DE genes from FTY720 versus blank comparison from the healing mucosal tissue, contained GO terms related to leukocytes and specifically neutrophils. The two neutrophil-related nodes were (1) GO:0030593-Neutrophil Chemotaxis and (2) GO:1990266-Neutrophil Migration, and within these two nodes, there were 9 genes differentially upregulated (C5ar1, Ccl2, Ccl7, Ccl9, Csf3r, Itgam, Nckap1l, Pf4, and Vav1) (Fig. 4). The 2 specific nodes are derived from the hierarchical GO tree, the root GO:0002376-Immune System Process as well as GO:0030595-Leukocyte Chemotaxis and GO:0050900-Leukocyte Migration. In addition, to the immune system-related nodes, GO terms related to Wound Healing were also identified (Supplemental Figure 1). The term GO:0009611-Response to Wounding is derived from the term GO:0006950-Response to Stress, and 22 genes from our differentially expressed genes were associated with this term (5430416N02Rik, 5830416P10Rik, Ano6, Bcl2, Ccl2, Ccr2, Cflar, Elk3, F10, F3, Gap43, Gm10791, Mapk8ip3, Mir17hg, Neat1, Pdpn, Pf4, Scnn1b, Snhg17, Syt11, Syt7, and Timp1). The heatmap and log2foldchange for these genes are represented in Figs. 9 and 10.

### FTY720 scaffolds increases protein expression levels of Vav1 during ONF healing

**Fig. 5:**
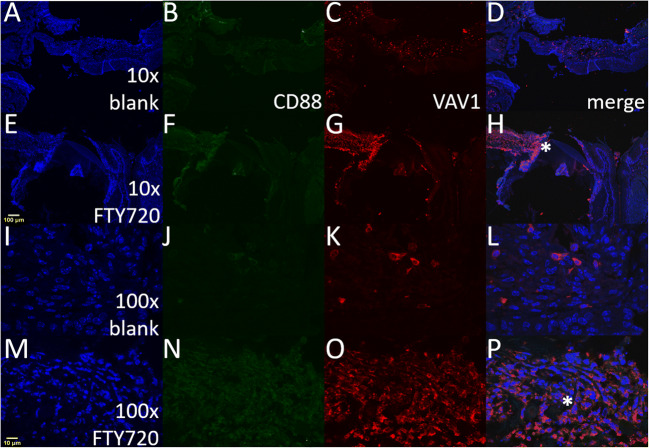
FTY720 induces protein expression of Vav1 at the site of the wound healing after palatal injury. Representative immunofluorescence images of mice palates 3 days post-injury stained against two important immunomodulators, Vav1 and CD88 proteins. (a–d) and (i–l) panels are from injured palate treated with blank nanofiber scaffold (vehicle). (e–h) and (m–p) panels are from injured palate treated with FTY720 nanofiber scaffold. The panels (a, e, i, and m) are nuclear staining using DAPI (blue). Panels (b, f, g, and n) are staining against CD88-Alexa488 (green). Panels (c, g, k, and o) are staining against Vav1-Alexa568 (red). Panels (d, h, l, and p) are the merged images. The asterisk symbol signalizes the same region in the injured palate treated with FTY720 between ×10 (H) and ×100 (P) panels. The images were acquired using the ×10 and ×100 objective on an Olympus F1000 microscope. The scale bars are noted in yellow, 10 μm for ×10 (A) and 100 μm for ×100 (M) images

To verify if genes upregulated by FTY720 treatment from inflammatory immune response pathways reflect the protein expression levels, mice palates 3 days post-injury were stained against two important immunomodulators, Vav1 and CD88 proteins. Vav1 is a guanine nucleotide exchange factor for Rho family GTPases, and it is widely expressed in all hemopoietic cells with an important function in the development and activation of several inflammatory cells (33). Cd88, also known as complement component 5a receptor 1 (C5ar1), is a G protein-coupled receptor for C5a. C5a has a chemotactic effect on granulocytes, monocytes, and macrophages. Furthermore, C5a has been shown to induce upregulation of adhesion molecules on neutrophils, and is thus also one of the factors responsible for neutrophil adhesion to endothelial cells (34). Confocal images from the mucosal defect tissue treated with FTY720 scaffolds (Fig. 5) showed increased expression of Vav1 at the edges of the wounded palate, but there was only a slight increase in expression of CD88 (Fig. 5f).

### Delivery of FTY720 nanofiber induced a heterogeneous neutrophil immunophenotypes at the site of ONF

**Fig. 6:**
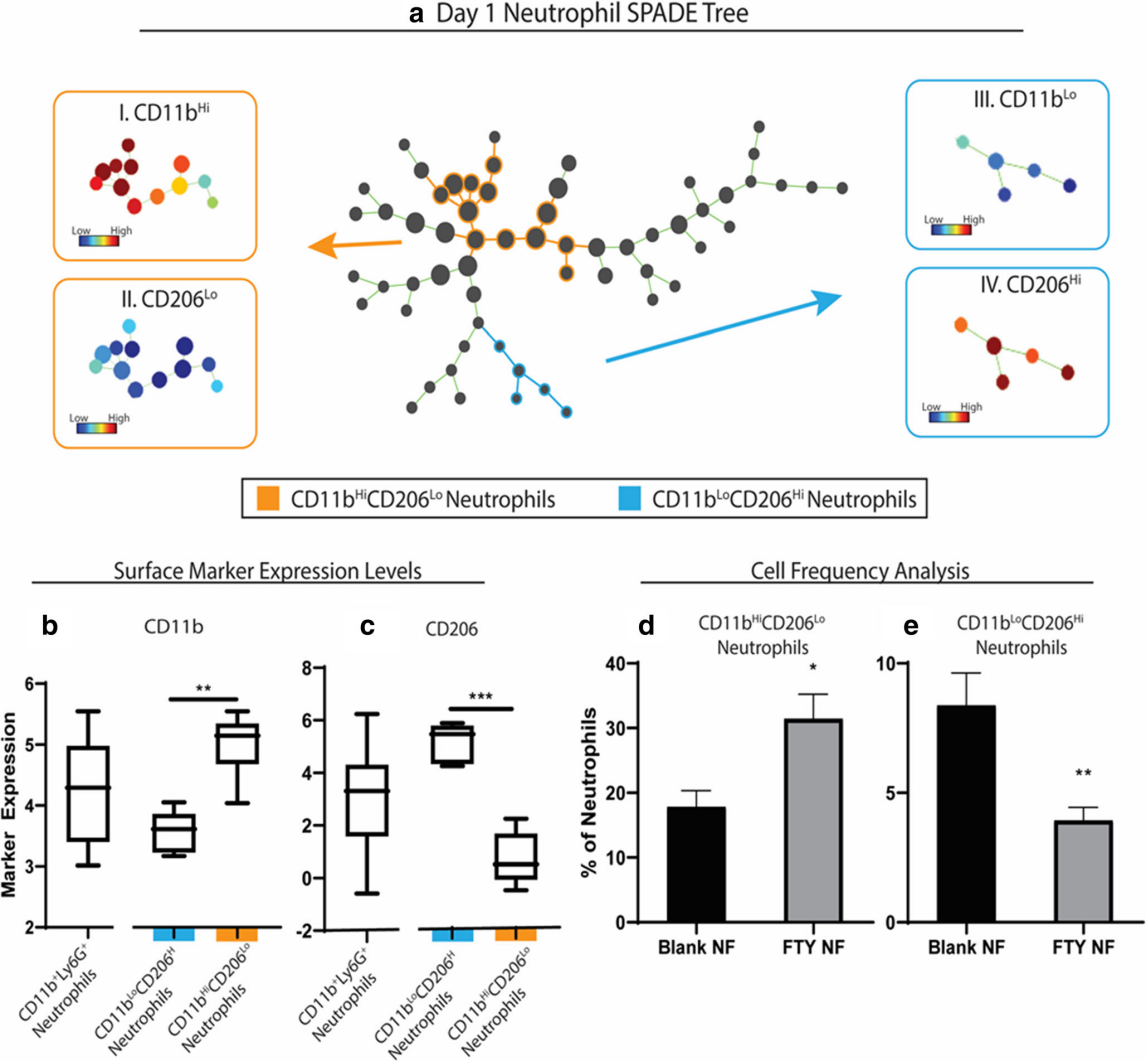
Subpopulations of neutrophils induced at day 1 post-injury by FTY720 during healing of wounded palate. A traditional gating strategy in which live, single cells were analyzed using CD11b+ as a general myeloid cell marker and Ly6G+ within the CD11b+ population as a neutrophil marker was used to visualize neutrophil heterogeneity in the myeloid cell compartment. Spade dendrogram constructed from neutrophils shows two subsets (colored in blue and orange) affected by FTY treatment (a). The corresponding surface marker expression heatmap (a, I-IV) and box and whisker plot are shown for each subset (a–c). The cell frequency of each neutrophil subset as a percentage of total neutrophils is shown (d, e). Statistical analyses were performed using two-tailed t-tests, *p < 0.05, **p < 0.01, ***p < 0.001, n = 5-6 animals per group

**Fig. 7:**
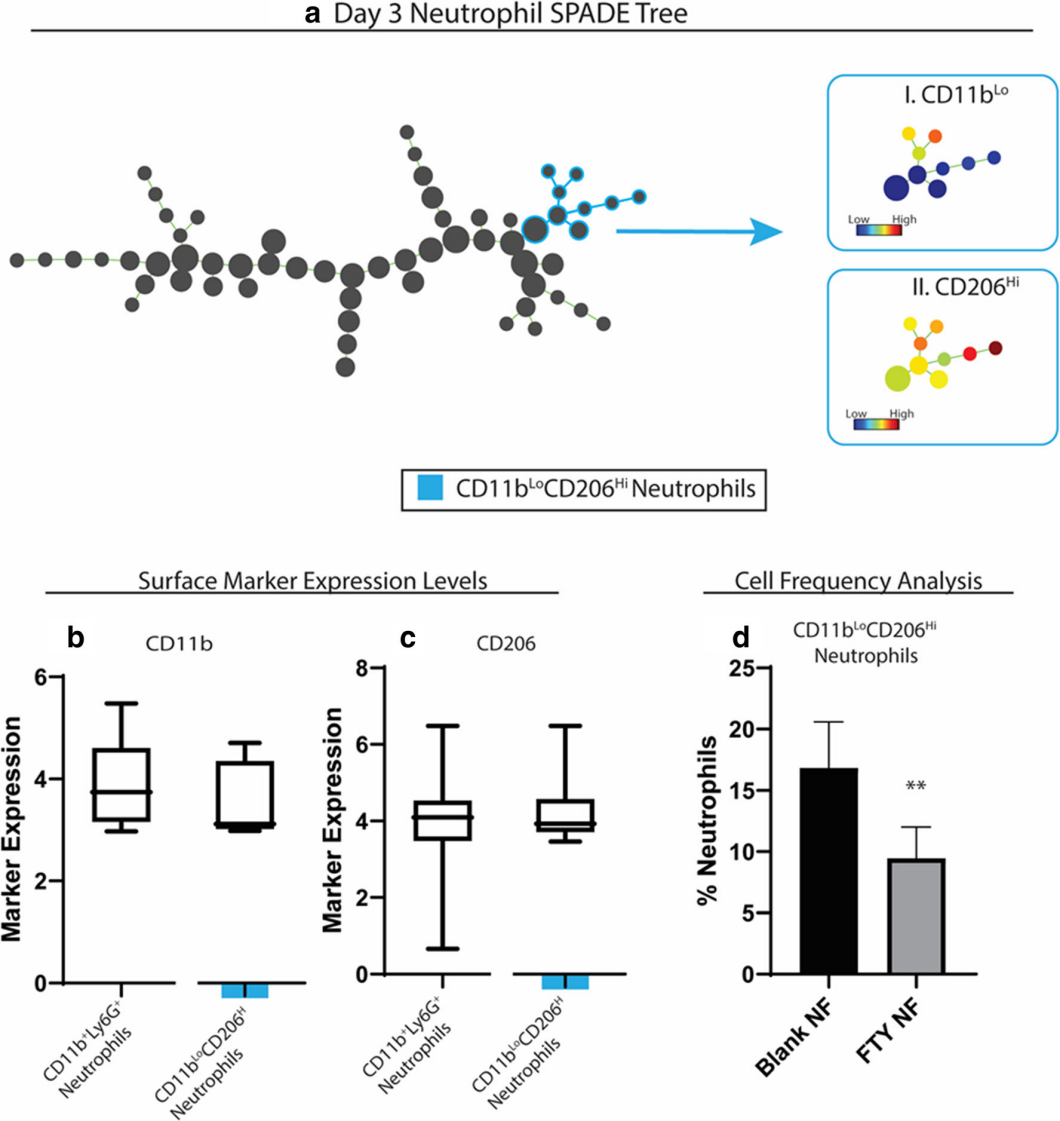
subpopulations of neutrophils induced at day 3 post-injury by FTY720 during healing of wounded palate. A traditional gating strategy in which live, single cells were analyzed using CD11b+ as a general myeloid cell marker and Ly6G+ within the CD11b+ population as a neutrophil marker was used to visualize neutrophil heterogeneity in the myeloid cell compartment. Spade dendrogram constructed from neutrophils shows one subset (colored in blue) affected by FTY treatment (a). The corresponding surface marker expression heatmap (a, I-IV) and box and whisker plot are shown for the subset (a–c). The cell frequency of the neutrophil subset as a percentage of total neutrophils is shown (d). Statistical analyses were performed using two-tailed t-tests, *p < 0.05, **p < 0.01, ***p < 0.001, n = 5-6 animals per group

Appreciation of the substantial heterogeneity of neutrophils and their roles in wound healing is rapidly increasing (35). We utilized SPADE to identify neutrophil subpopulations of cells that are significantly affected by FTY720 delivery. SPADE is a novel pseudotime analysis algorithm that is able to reconstruct complex cellular hierarchies of immune cell transitions in order to reveal rare cell states based on a limited set of surface markers (16). We constructed SPADE dendrograms of CD11b^+^Ly6G^+^ neutrophils at both day 1 and day 3 post-injury. This analysis allows us to delve into the heterogeneity of neutrophils and how FTY720 treatment affects specific subpopulations. At day 1 post-injury and nanofiber implantation, SPADE identified two populations of cells that were significantly affected by FTY720 treatment when compared to blank nanofibers (Fig. 6). Phenotypes were then assigned visually to larger clusters by overlaying the expression of relevant surface markers. Two key subpopulations were identified, including 1) a subpopulation (colored in orange) with high expression of CD11b (Fig. 6a, I) and low expression of CD206 (Fig. 6a, II) and 2) a second subpopulation (colored in blue) with low expression of CD11b (Fig. 6a, III) and high expression of CD206 (Fig. 6a, IV). Quantification of neutrophil subsets confirmed that FTY720 scaffold treatment resulted in a significant increase in the accumulation of CD206^lo^ neutrophils compared to vehicle controls at day 1. SPADE analysis at day 3 marked a shift in neutrophil subset response with a relative decrease in CD206^lo^ expressing neutrophils (Figs. 7a–d). Analysis of the immune subsets suggests that this temporal shift is targeted to the aforementioned subsets and no significant changes on the other subpopulations of neutrophils (Supplemental figures 2 and 3). These novel observations of heterogeneous neutrophil subset populations in the oral mucosa suggest a complex and underappreciation role for neutrophil in the therapeutic program of FTY720.

## Discussion

Our group has previously shown that local and not systemic delivery of FTY720 to inflamed and ischemic tissues reduces pro-inflammatory cytokine secretion and increases pro-regenerative cytokine secretion (11). The altered balance of cytokine secretion results in preferential recruitment of anti-inflammatory monocytes from circulation. Recently, we also demonstrated that FTY720 delivery on a nanofiber scaffold following ONF creation leads to wound contracture at day 3 and healing at day 5 compared to the (blank) vehicle control scaffold (5). Evaluation of the monocyte and macrophage population during the ONF healing process revealed an increase in Ly6C^lo^ pro-regenerative monocytes and M2 macrophages in the wound bed compared to blank scaffolds, similar to results found in a cutaneous wound healing studies (5, 10). Kimball et al. also demonstrated independently that Ly6C^hi^ blood monocyte/macrophage induce chronic inflammation and impaired wound healing in murine model of Diabetes Mellitus (36). While some studies have evaluated the impact of FTY720 on monocytes and macrophages during cutaneous wound healing, little is known about how FTY720 delivery regulates gene transcription during ONF wound healing. In this manuscript, we focused on the early impact of FTY720 delivery on gene transcription, focusing on the inflammatory phase of wound healing using KEGG and GO pathway analysis and coupled that with flow cytometric evaluation. We described for the first time the inflammatory gene expression induced by FTY720 in the injured palate mucosa and therapeutic modulation of neutrophil subset recruitment induced by FTY720.

Specifically, we used a murine model of ONF formation to study the changes in gene transcription induced by FTY720 nanofiber scaffolds delivered at the site of injury by RNA sequencing analysis. Das et al. showed previously that FTY720 regulated key inflammatory genes in LPS-treat microglia cells by mRNA sequencing (37). Analysis of palate mucosa injured tissue identified 669 genes that were differentially regulated during the first 3 days of ONF wound healing after FTY720 scaffold delivery. As FTY720 acts on multiple S1P receptors, we confirmed that the sphingolipid signaling was activated by the presence of FTY720 loaded scaffold in the mucosa (Fig. 2). These results further validate the bioactivity and specificity of S1P receptor-targeted drugs delivered from nanofiber scaffolds to activate the sphingolipid signaling pathway. One of the major effectors of this pathway is the PI3kinase signaling, and results show that phosphatidylinositol-4,5-Bisphosphate 3-Kinase Catalytic Subunit Delta (Pi3kcd) was upregulated in our model by FTY720. In a mouse model of multiple sclerosis, RNA sequencing was performed to determine the effects of FTY720 on B cell-relevant gene expression, and similar to our results, genes from PI3K pathway were differentially regulated, including Ikbkb, Pi3kaq1, and Vav3 (38). Phosphoinositide 3-kinases (PI3K), which is a dimeric enzyme, consisting of a 110 kD catalytic subunit gamma and a regulatory subunit of either 55, 87, or 101 kD phosphorylate inositol lipids and are involved in the immune response (39, 40). The protein encoded by this gene is a class I PI3K found primarily in leukocytes. Phosphoinositide-3-Kinase Regulatory Subunit 5 (Pi3kr5) subunit was also upregulated by FTY720. Pi3kr5 gene encodes the 101kD regulatory subunit of the class I PI3K gamma complex. The direct effectors of the PI3K signaling sit within a complex network that crosstalk with several other pathways (RhoA GTPases, Oxidative Stress, Integrins, Chemokines, and Cytokines signaling) and consequently are responsible for important neutrophil functions such as adhesion, chemotaxis, secretion, and oxidative stress (41, 42). In addition to the Pi3ks genes, other important genes from the sphingolipid related signaling pathway were found, stress fiber and migration genes were increased: Plcb2 (Phospholipase C Beta 2), Prkcb (Protein Kinase C Beta), and Rock1-2 (Rho Associated Coiled-Coil Containing Protein Kinase 1-2) (43–45). Together, the data presented in this manuscript demonstrate that FTY720 nanofiber scaffold locally delivery increases the downstream gene expression of the sphingosine signaling pathway as well as genes critical to cell mobility and migration of immune cells.

We also evaluated 669 differentially expressed genes following FTY720 scaffold treatment of ONF wounds using the KEGG Database. The local delivery of FTY720 scaffold in the ONF induced the expression of chemokines, cytokine receptors, and ligands genes, confirming its role in the chemotaxis, migration, and activation of leukocytes (46, 47). We specifically identified inflammatory immune pathways map04062-Chemokine signaling pathway, map04060-Cytokine-cytokine receptor interaction, and map04670-Leukocyte transendothelial migration (Fig. 3). Leukocyte migration from the blood into the tissues via transendothelial migration is an essential step during the inflammation process. During this diapedesis of leukocytes, the leukocytes bind to endothelial cell adhesion molecules (CAM) and then migrate across the vascular endothelium (31, 48). Several fundamental genes in this process were upregulated in the ONF mucosa following the local delivery of FTY720, including Vav1, Rock1, Rock2, Itgam, Ncf2 (p67phox), and Ncf4 (p47phox). Several publications have shown the importance of these genes in wound healing response. Vav3^−^/^−^ and Vav1^−^/^−^; Vav3^−^/^−^ mice showed significantly delayed healing of full-thickness excisional wounds (49). It has also been shown that the Vav1-Rac1 pathway plays an important role in the formation of lamellipodia in foam cells (50). Enhancing mesenchymal stem cells with stearic acid methyl ester accelerated cartilage regeneration through the Vav1/Rock2 signaling pathway (51). Rho-associated coiled-coil kinases (ROCKs) are key regulators of cytoskeletal rearrangement. Sobel et al. showed that FTY720-P selectively activated the Gα12/13/Rho/ROCK pathway via the S1P2 receptor in myofibroblasts. Activation of that pathway by FTY720-P caused potent myofibroblast contraction similar to that induced by the natural ligand S1P (52).

Oxidative stress plays a critically important role during wound healing (53). One of the most important sources of intracellular reactive oxygen species (ROS) is the enzyme NADPH oxidase (Nox). NADPH oxidase enzyme complex is formed by Nox and other cytosolic subunits (p67phox, p47phox, p22phox, and poldip2) and catalyzes the production of ROS from molecular oxygen (54). In Fig. 3, we demonstrated that several of the Nox cytosolic subunits (p67phox, p47phox) were upregulated following FTY720 nanofiber scaffold delivery. Nox1 and its derived reactive oxygen species are suggested to regulate inflammation, cell proliferation, migration, and extracellular matrix synthesis, which contribute to the processes of tissue injury and repair (55). However, it has been shown that excessive ROS is deleterious to the healing. S42909, a potent NADPH oxidase activity inhibitor, improved the healing process by dampening excessive inflammation and facilitating collagen deposition without wound contraction (56). Roy et al. demonstrated the role of H_2_O_2_ in wound healing in vivo; in this study, they showed that at the wound site, low concentrations of H_2_O_2_ supported the healing process, especially in p47(phox)- and MCP-1-deficient mice in which endogenous H_2_O_2_ generation is impaired (57). Overall, the data from our study suggest that the FTY720 scaffold induced the ROS pathway via the increased expression of Nox subunits (p67phox, p47phox).

Gene Ontology analysis of our 669 DE genes, from the injured palate treated with FTY720 scaffold compared to the blank scaffold, using GOnet application generated an interactive Gene Ontology analysis graph. The analysis identified several key immune system nodes and the respective differentially expressed genes (Fig. 4). In this graph, multiple nodes are related to neutrophil biology (GO:0030593-Neutrophil Chemotaxis and GO:1990266-Neutrophil Migration). One of the genes identified in this analysis is complement component 5a receptor 1 (C5ar1) or CD88 (Cluster of Differentiation 88), a receptor for the chemotactic and inflammatory peptide anaphylatoxin C5a. C5ar1 stimulates chemotaxis, granule enzyme release, intracellular calcium release, and superoxide anion production (58, 59). Another gene identified that regulates neutrophil function is Colony Stimulating Factor 3 Receptor (Csf3r) also known as CD114 (Cluster of Differentiation 114). The importance of Csf3r lays on its crucial role in the proliferation, differentiation, and survival of cells along the neutrophilic lineage (32, 60). Integrin alpha M (Itgam) also known as CD11b (cluster of differentiation molecule 11B) is also upregulated by FTY720 delivery, and it has an important role in the adherence of neutrophils and monocytes to endothelium (60). These data together showed that FTY720 plays an important role in the fine modulation of the neutrophil biology in the oral wound process.

**Fig. 8:**
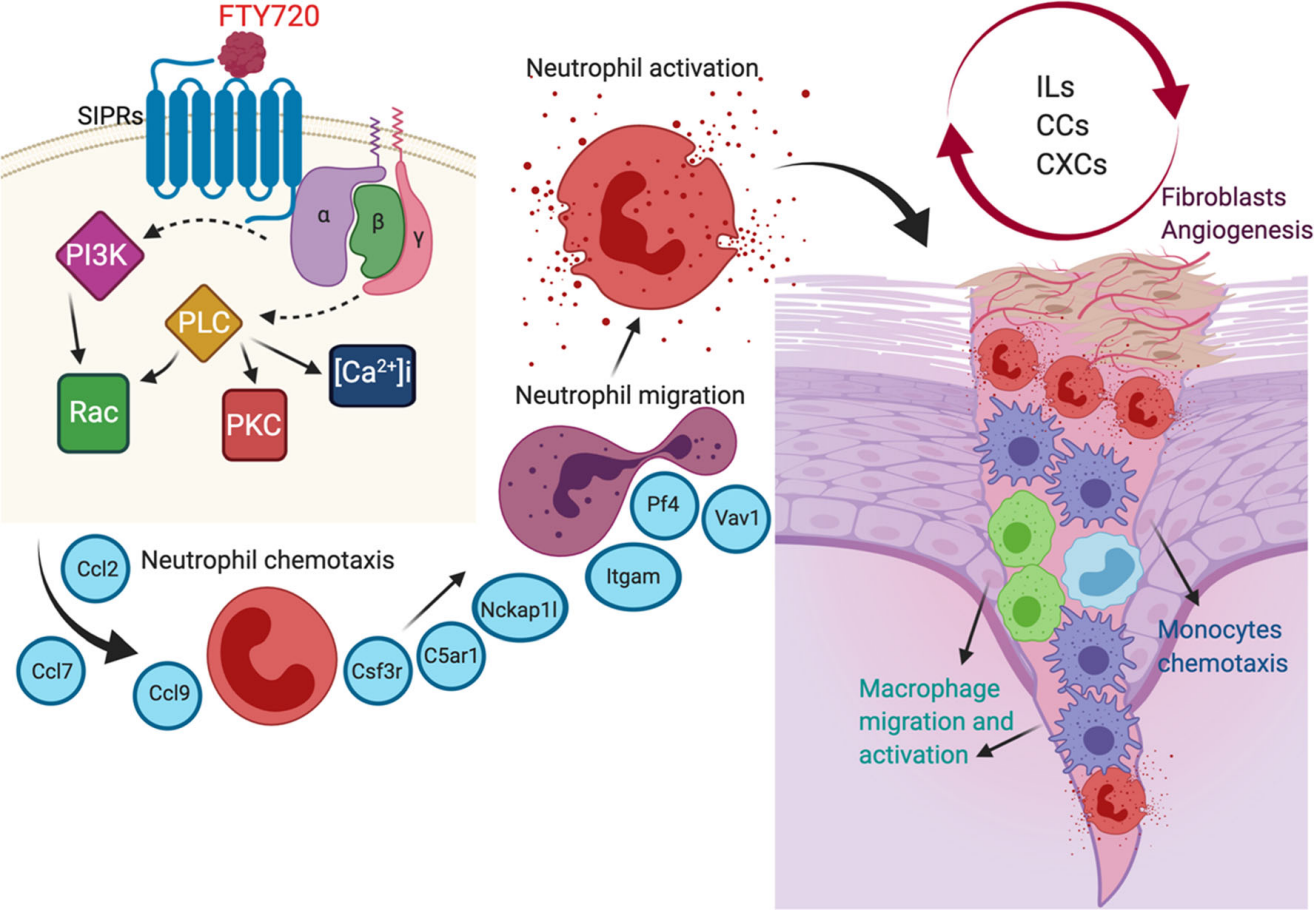
Schematic representation of the role of neutrophils during wound healing process after palatal injury. Schematic representation of mechanisms FTY720 signaling accelerates the wound healing response

The wound healing process involves 3 major phases, inflammation, proliferation, and remodeling/scar formation (27). The neutrophils are the first responders of the immune system in response to injury or infection. One of the important functions of the neutrophils in the wound is to secrete inflammatory mediators that will induce chemotaxis and activation of mononuclear phagocytes, including monocytes and macrophages. Neutrophils are also capable of generating other factors that induce resolution of inflammation, induce angiogenesis, and remodeling of extracellular matrix by fibroblasts (Fig. 8). We previously demonstrated that delivery of FTY720 nanofiber scaffolds following ONF creation increased LY6C^lo^ monocytes and pro-regenerative M2 macrophages (5). Here, we demonstrated that the delivery of FTY720 scaffolds immunomodulate the ONF wound healing response by first controlling local gene expression of neutrophil subsets.

**Fig. 9:**
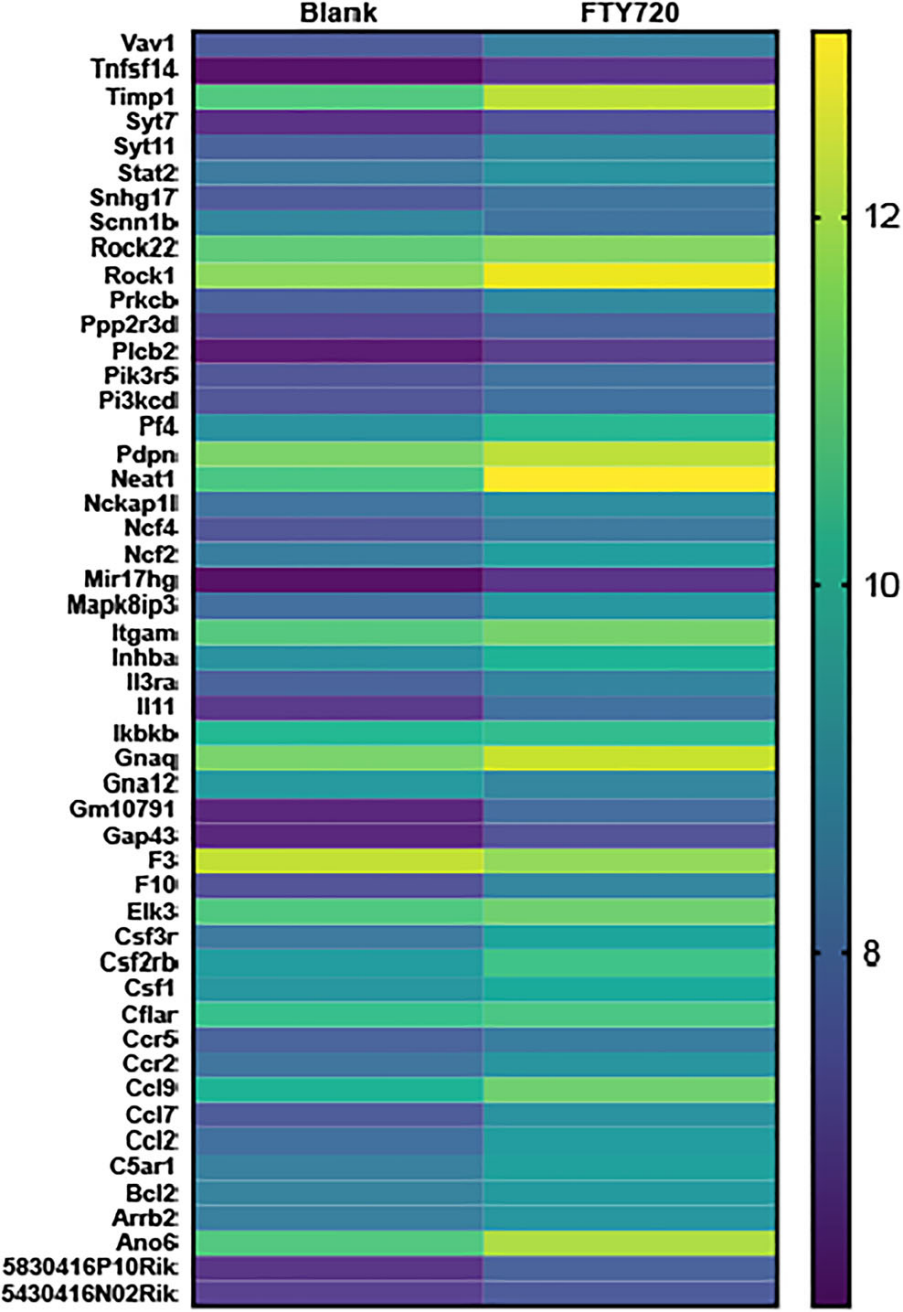
– Heatmap of the DE genes.

**Fig. 10:**
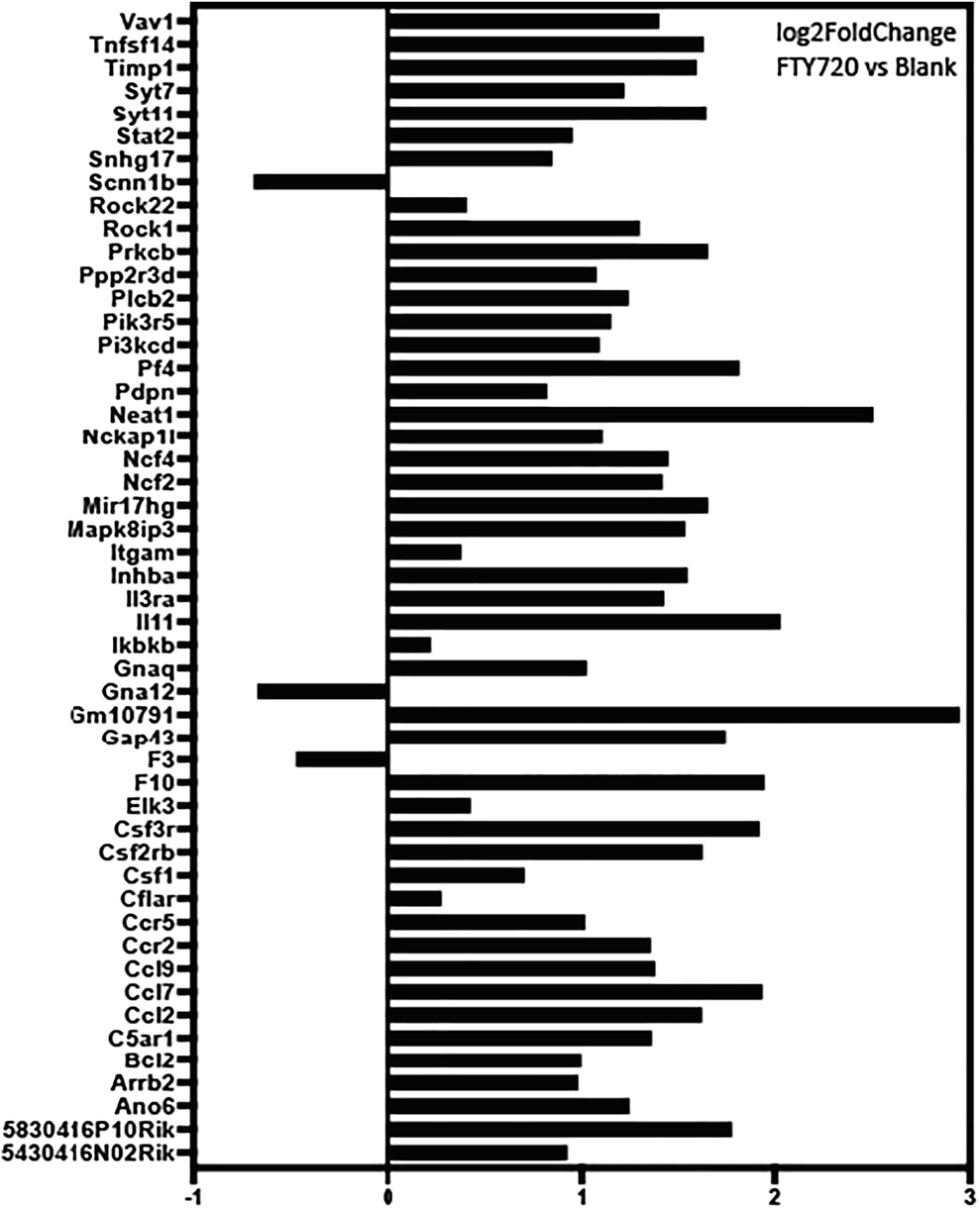
- Log2foldchange (FTY720 versus Blank) of the DE genes.

Current research in the field of neutrophil biology has revealed that neutrophils possess a highly diverse repertoire of functional responses (35, 61). The emergence of functional neutrophil phenotypes opens up the possibility to harness the therapeutic potential of neutrophil subsets. However, classical single-cell analytical techniques such as sequential bi-plot gating are unable to quantify non-traditional immune populations. To overcome these challenges, we employed SPADE, a dimensionality reduction and pseudotime analysis technique, that allowed analysis of neutrophil heterogeneity in response to FTY720 delivery in a nanofiber implantation after an ONF. Using SPADE, we found two distinct neutrophil subpopulations that were significantly affected by FTY720 treatment. Interestingly, the subpopulation of neutrophils that had an increased frequency with FTY720 treatment has a high expression of CD11b, as it was also seen in our Gene Ontology data, and low expression of CD206, which is a common marker for alternatively activated macrophages and may similarly marker a relevant phenotypic neutrophil subset (62). Our research suggests that improved healing outcomes and reduced ONF formation shown in our previous research are preceded by modulation of the early neutrophil immune response. These data also suggest that sustained, local delivery of FTY720 using biomaterials results in an accumulation of targeted neutrophil subsets. The prospect of increasing the frequency of more highly active neutrophils as well as enhancing phagocytic activity, which is vital to maintain health and regenerative cavity in the oral mucosa, is especially intriguing and warrants further investigation into the broader surgical implications in human wound healing during cleft palate surgical repair.

## Supplementary Information

Supplemental Figure 1 - FTY720 induces wound healing response after palatal injury. Gene Ontology analysis graph. GOterm analysis of data obtained from sequencing of RNA that was isolated from palate mucosa treated immediately after injury with or without FTY720 scaffolds showing term related with Wound Healing. Highlighted in yellow is the correspondent node GO:0009611 - Response to Wounding. In the left on the graph is the list of genes present in this node. (PNG 167 kb)

High Resolution Image (TIF 1964 kb)

Supplemental Figure 2 – Subpopulations of neutrophils at day 1 post injury during healing of wounded palate. A traditional gating strategy in which live, single cells were analyzed using CD11b+ as a general myeloid cell marker and Ly6G+ within the CD11b+ population as a neutrophil marker was used to visualize neutrophil heterogeneity in the myeloid cell compartment (PNG 392 kb)

High Resolution Image (TIF 2220 kb)

Supplemental Figure 3 - Subpopulations of neutrophils at day 3 post injury during healing of wounded palate. A traditional gating strategy in which live, single cells were analyzed using CD11b+ as a general myeloid cell marker and Ly6G+ within the CD11b+ population as a neutrophil marker was used to visualize neutrophil heterogeneity in the myeloid cell compartment (PNG 342 kb)

High Resolution Image (TIF 2190 kb)

Supplemental Table 1 – KEGG Pathways of the DE genes. (PNG 256 kb)

High Resolution Image (TIF 952 kb)

Supplemental Table 2 – Nomenclature of the DE genes. (PNG 191 kb)

High Resolution Image (TIF 1021 kb)
