# Supplementary figures and images for: Local delivery of FTY720 induces neutrophil activation through chemokine signaling in an oronasal fistula model
Source: Regen Eng Transl Med. 2021 May 13;7(2):160–74. doi: 10.1007/s40883-021-00208-z (PMC8549964; doi:10.1007/s40883-021-00208-z)

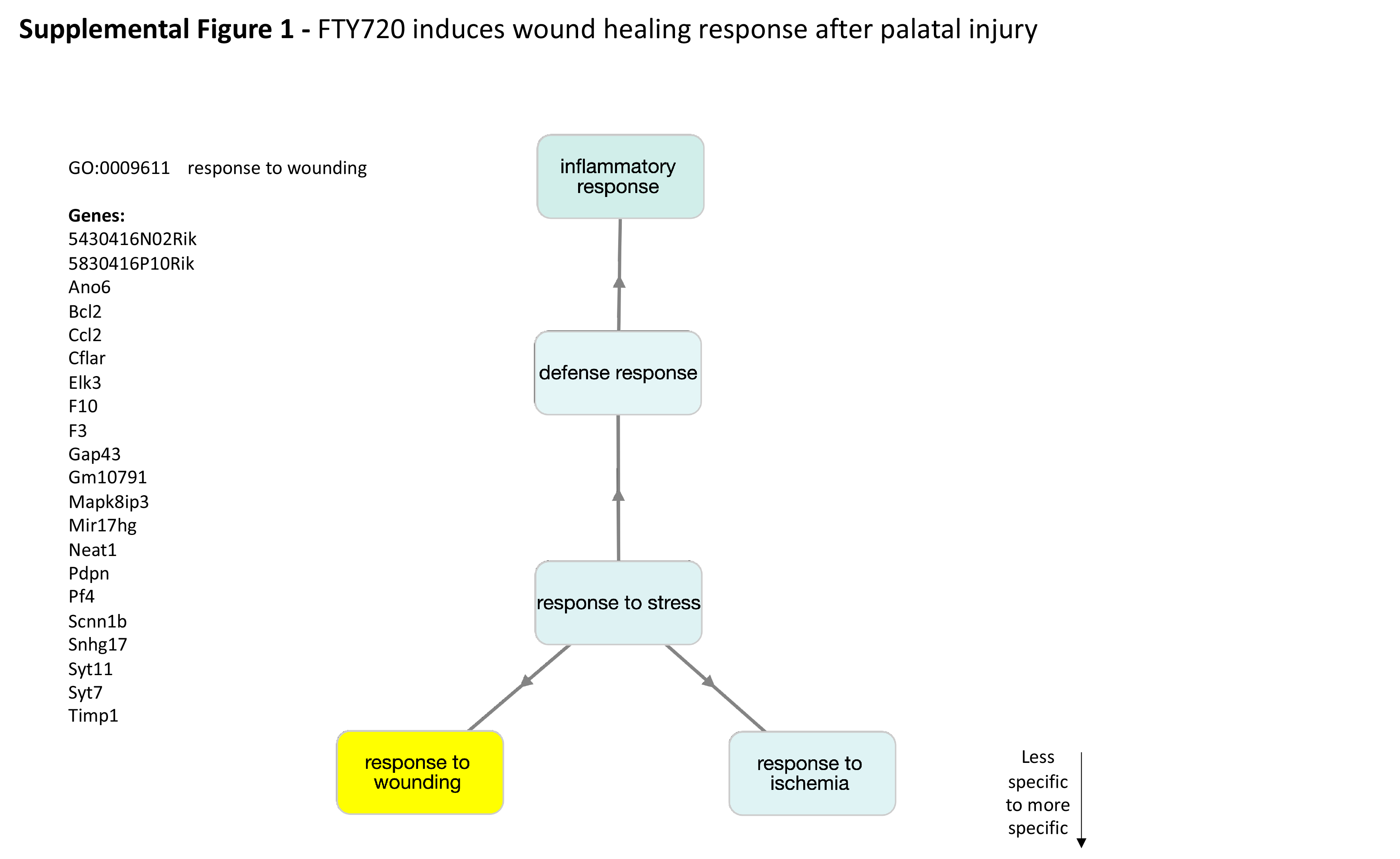

Supplement: Supplementary file 1 — - FTY720 induces wound healing response after palatal injury. Gene Ontology analysis graph. GOterm analysis of data obtained from sequencing of RNA that was isolated from palate mucosa treated immediately after injury with or without FTY720 scaffolds showing term related with Wound Healing. Highlighted in yellow is the correspondent node GO:0009611 - Response to Wounding. In the left on the graph is the list of genes present in this node. (PNG 167 kb) [file 40883_2021_208_Fig9_ESM.png]

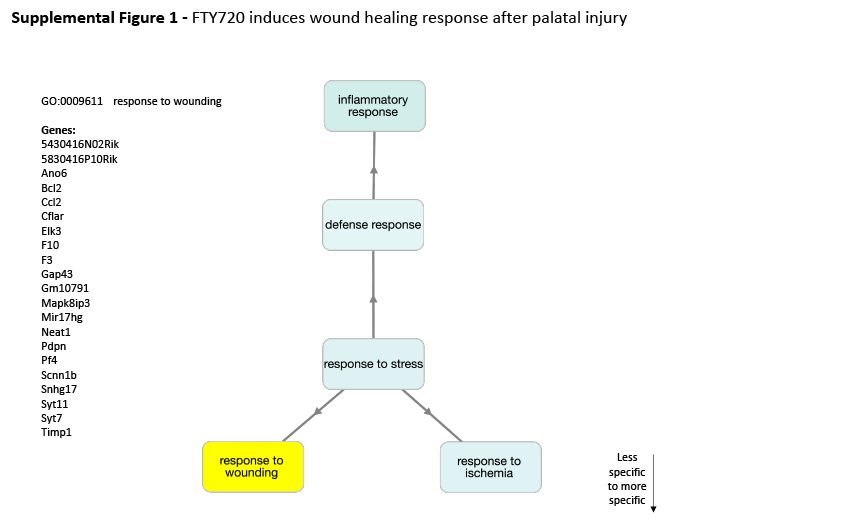

Supplement: Supplementary file 2 — High Resolution Image (TIF 1964 kb) [file 40883_2021_208_MOESM1_ESM.tif]

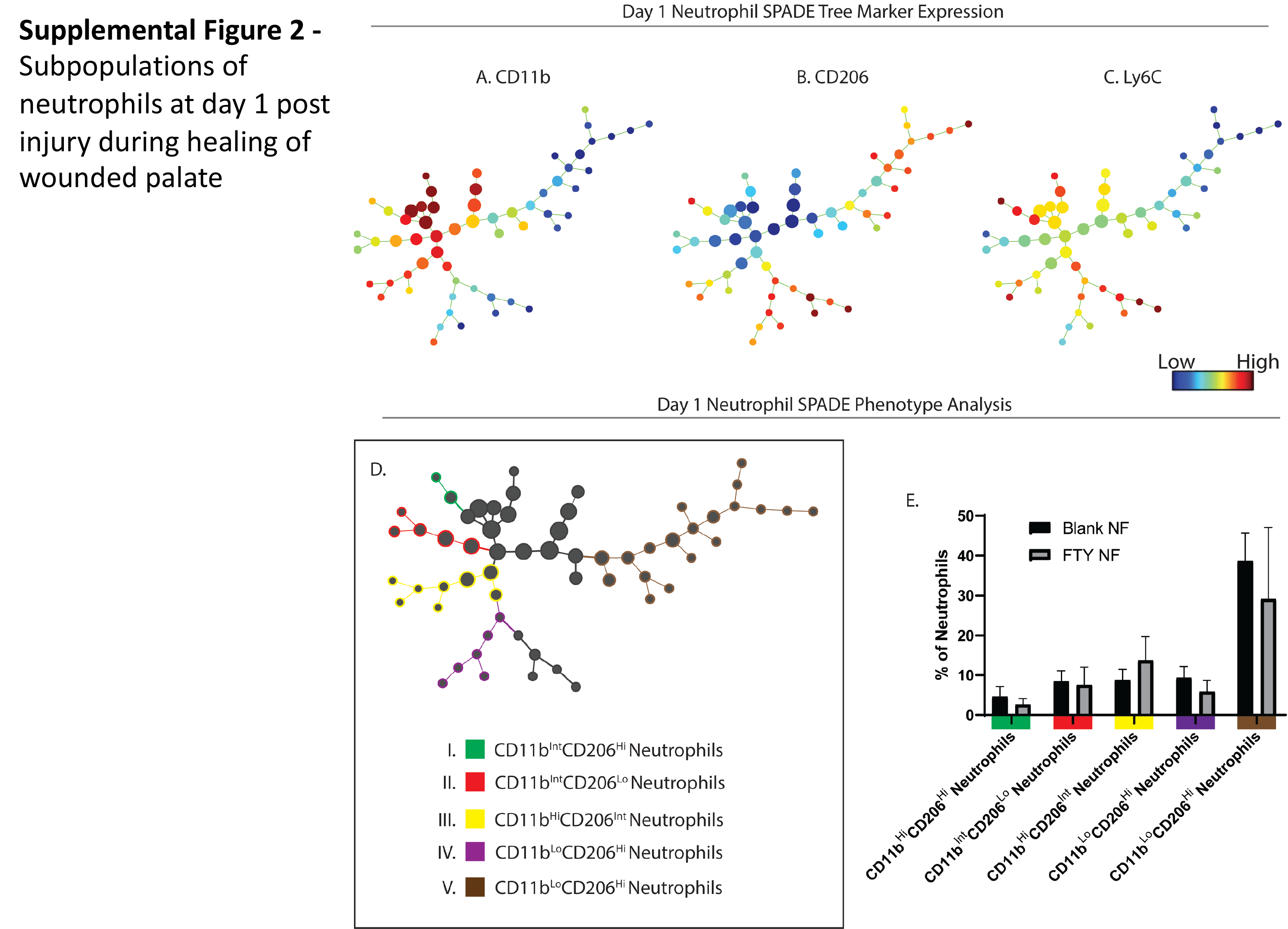

Supplement: Supplementary file 3 — – Subpopulations of neutrophils at day 1 post injury during healing of wounded palate. A traditional gating strategy in which live, single cells were analyzed using CD11b+ as a general myeloid cell marker and Ly6G+ within the CD11b+ population as a neutrophil marker was used to visualize neutrophil heterogeneity in the myeloid cell compartment (PNG 392 kb) [file 40883_2021_208_Fig10_ESM.png]

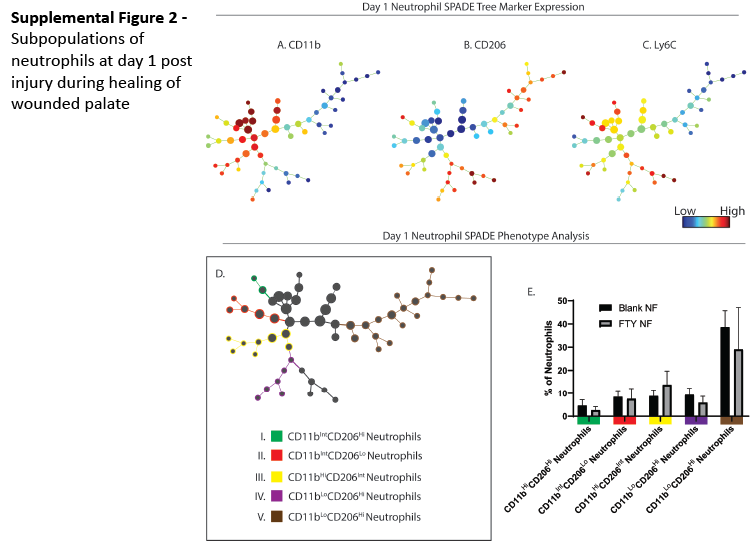

Supplement: Supplementary file 4 — High Resolution Image (TIF 2220 kb) [file 40883_2021_208_MOESM2_ESM.tif]

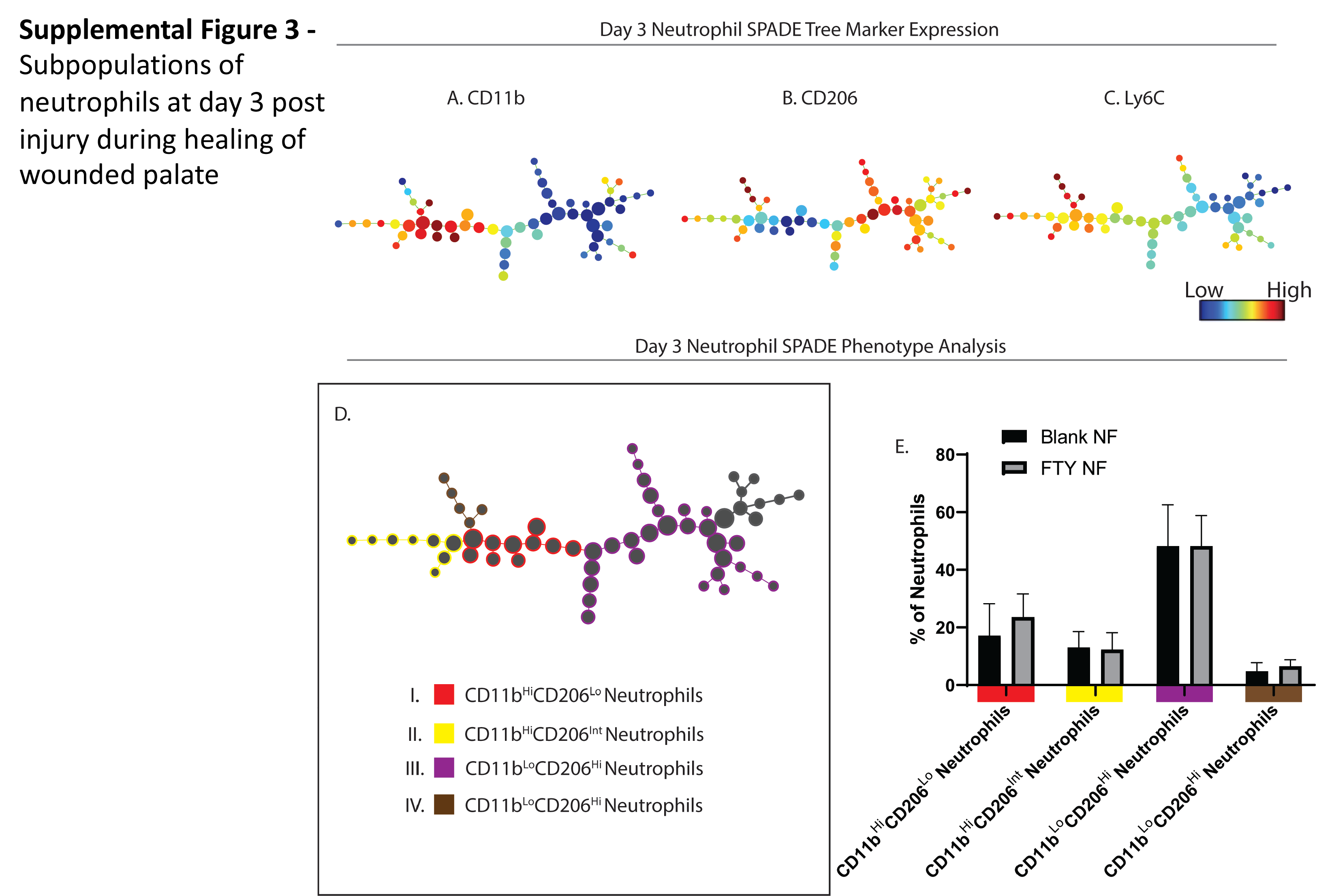

Supplement: Supplementary file 5 — - Subpopulations of neutrophils at day 3 post injury during healing of wounded palate. A traditional gating strategy in which live, single cells were analyzed using CD11b+ as a general myeloid cell marker and Ly6G+ within the CD11b+ population as a neutrophil marker was used to visualize neutrophil heterogeneity in the myeloid cell compartment (PNG 342 kb) [file 40883_2021_208_Fig11_ESM.png]

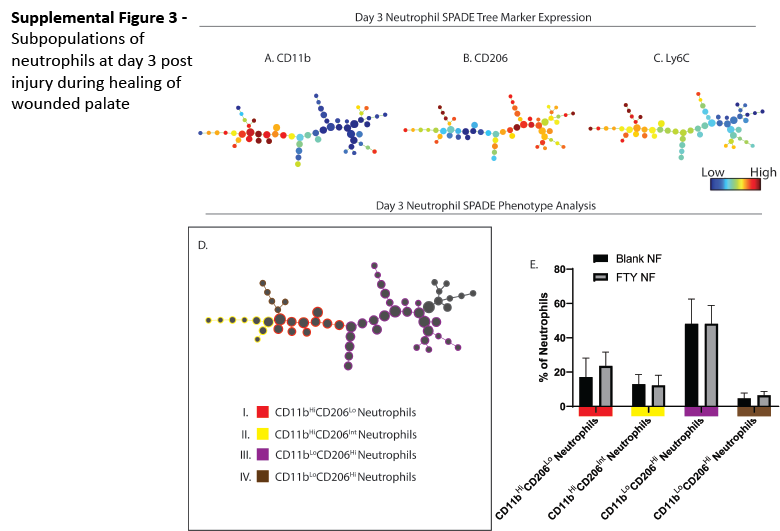

Supplement: Supplementary file 6 — High Resolution Image (TIF 2190 kb) [file 40883_2021_208_MOESM3_ESM.tif]

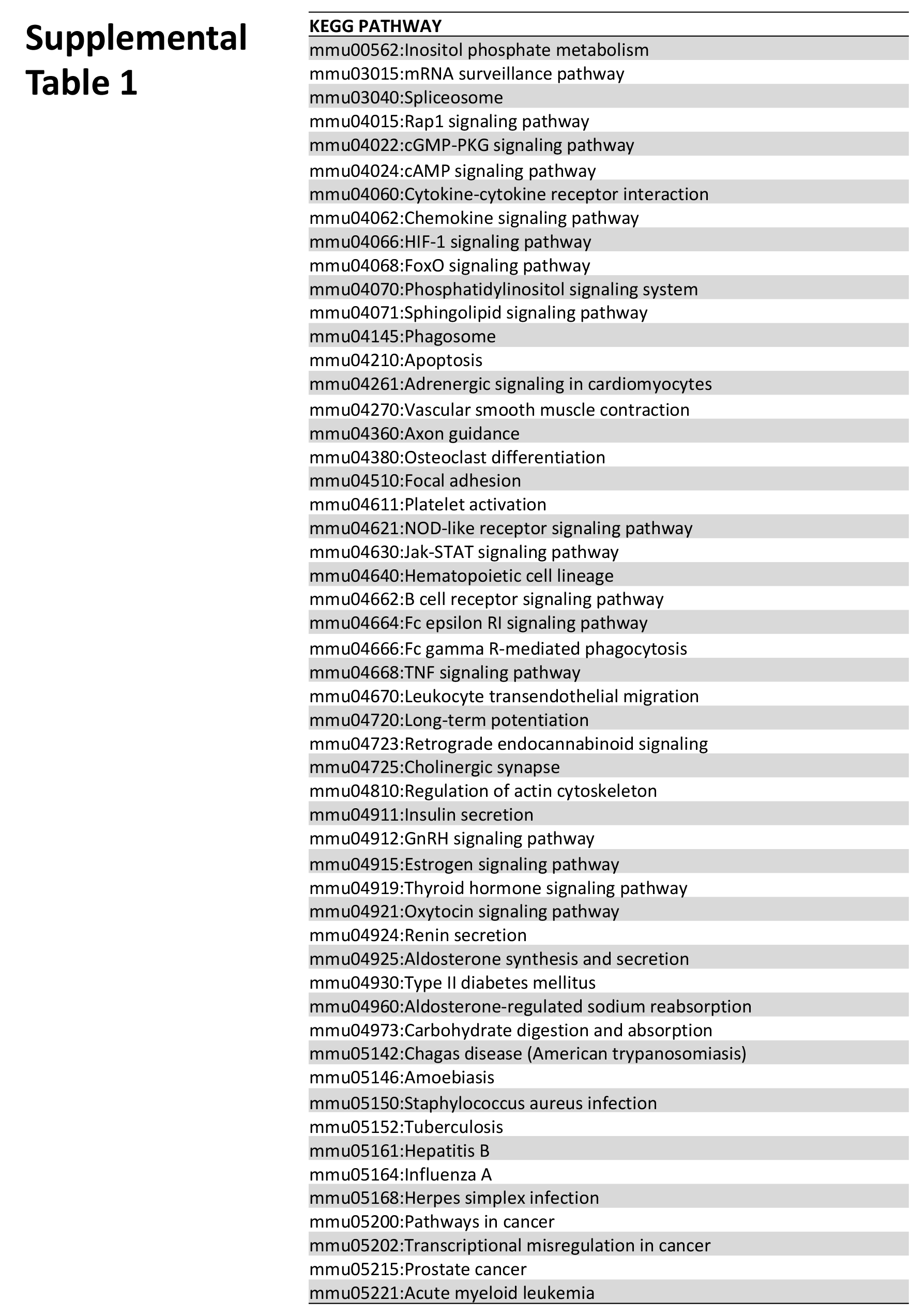

Supplement: Supplementary file 7 — – KEGG Pathways of the DE genes. (PNG 256 kb) [file 40883_2021_208_Fig12_ESM.png]

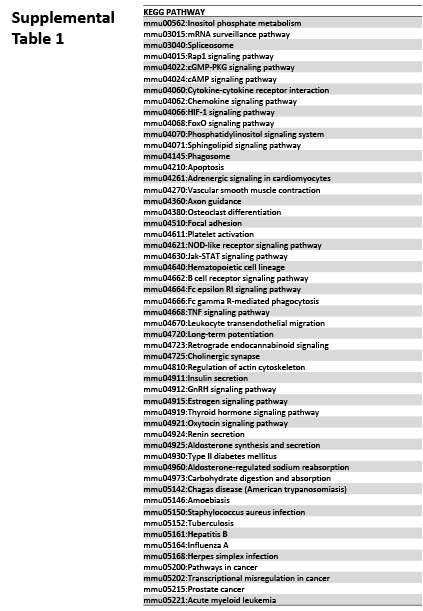

Supplement: Supplementary file 8 — High Resolution Image (TIF 952 kb) [file 40883_2021_208_MOESM4_ESM.tif]

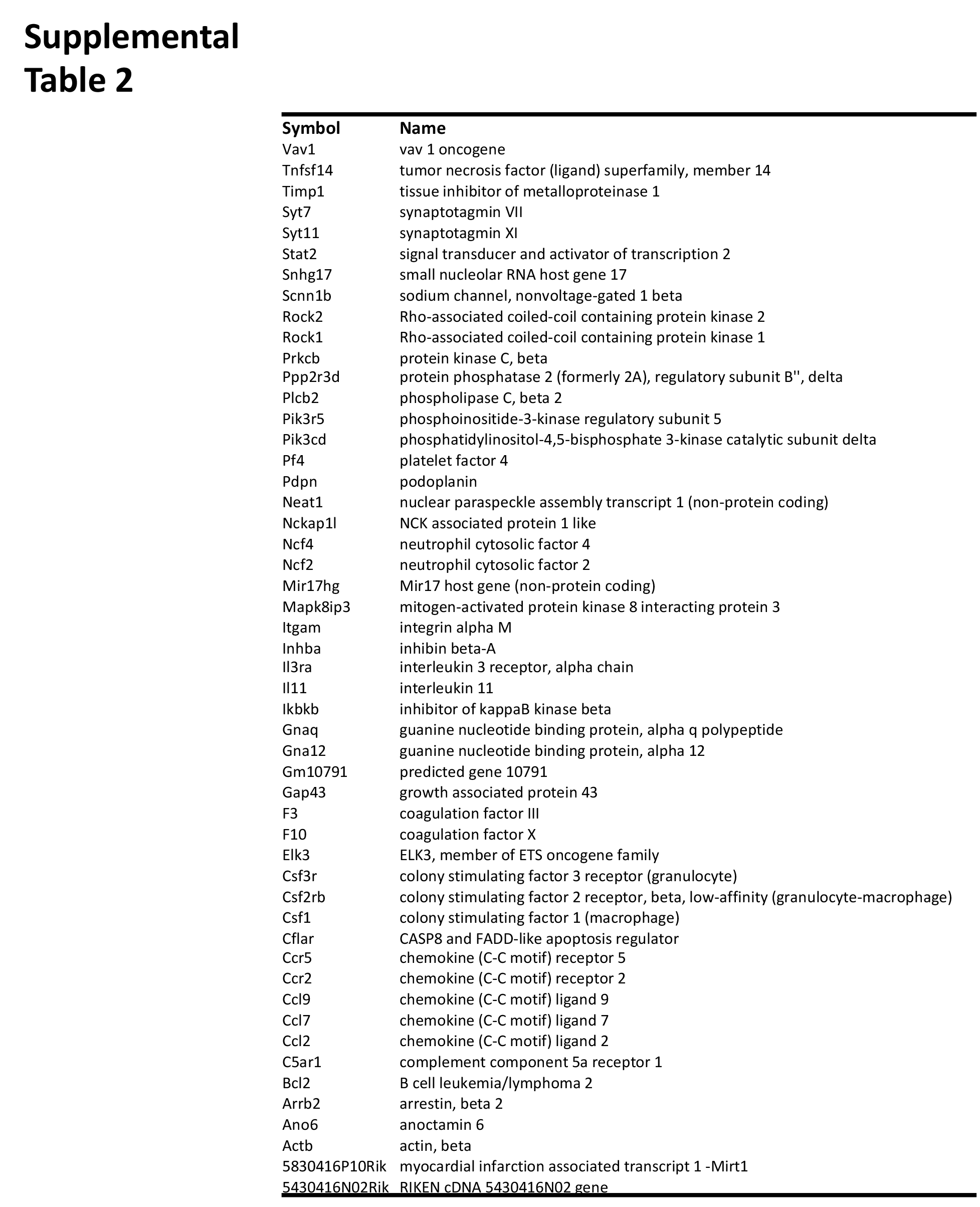

Supplement: Supplementary file 9 — – Nomenclature of the DE genes. (PNG 191 kb) [file 40883_2021_208_Fig13_ESM.png]

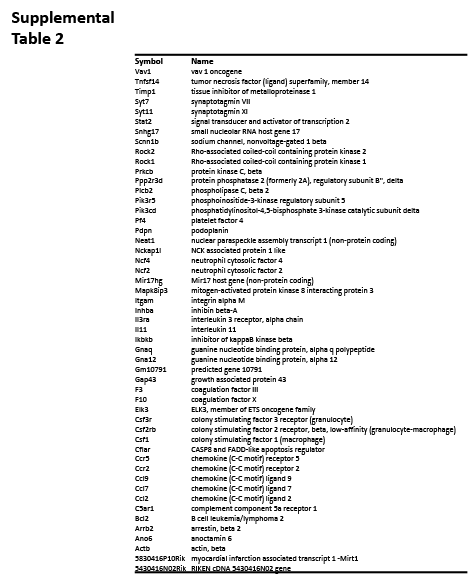

Supplement: Supplementary file 10 — High Resolution Image (TIF 1021 kb) [file 40883_2021_208_MOESM5_ESM.tif]
